# The occurrence of the multidrug resistance (MDR) and the prevalence of virulence genes and QACs resistance genes in *E. coli* isolated from environmental and avian sources

**DOI:** 10.1186/s13568-019-0920-4

**Published:** 2019-12-03

**Authors:** Mohamed E. Enany, Abdelazeem M. Algammal, Soad A. Nasef, Sara A. M. Abo-Eillil, May Bin-Jumah, Ayman E. Taha, Ahmed A. Allam

**Affiliations:** 1 Bacteriology, Mycology and Immunology Department, Faculty of Veterinary Medicine, Suez Canal University, Ismailia, 41522 Egypt; 2 Reference Laboratory for Veterinary Quality Control on Poultry Production, Animal Health Research Institute, Giza, Egypt; 3 Biology Department, College of Science, Princess Nourah Bint Abdulrahman University, P.O. Box 24428, Riyadh, 11671 Saudi Arabia; 4 Department of Animal Husbandry and Animal Wealth Development, Faculty of Veterinary Medicine, Alexandria University, Edfina, 22578 Egypt; 5 Department of Zoology, Faculty of Science, Beni-Suef University, Beni-Suef, 6521 Egypt

**Keywords:** *E. coli*, Chickens, Virulence genes, QACs resistant genes

## Abstract

Colibacillosis is a major disease affecting poultry leads to high morbidity and mortality which causing tremendous economic losses worldwide. These economic disparities are amplified among low and middle-income where sanitation and hygiene are challenged by the increasing demand for quality sources of animal protein. With a view to investigating the prevalence of virulence genes and QACs resistance genes as well as monitoring the antibiogram of *E. coli* strains, a total of 368 specimens were collected from diseased broiler chickens (n = 226) and environmental sources (n = 142) at large-scale poultry farms in Ismailia Governorate, Egypt. The bacteriological examination proved that *E. coli* prevalence was 26.76% and 50.44% in the farm environment and diseased broilers, respectively. In tandem, the isolated *E. coli* strains were serogrouped, determining the most common serotypes were O78, O1:H7, O91:H21 and O126. Isolates were tested for antimicrobial susceptibility against 12 antibiotics, screened for 4 virulence genes (*iss, papC, eaeA*, and *cfaI*), and screened for 3 QACs resistance genes (*qacEΔ1, qacA/B,* and *qacC/D*). All the tested strains were positive for *iss* and *papC* genes, only 20.3% of the tested strains were positive for *eaeA* gene, moreover, the examined strains were negative to *CFAI* gene. Furthermore, all the tested strains were positive for *qacEΔ1, qacA/B,* and *qacC/D* genes. In conclusion; virulence genes (*iss, papC*) as well as QACs resistance genes are common in avian Pathogenic *E. coli* and environmental strains and are mainly associated with multi-drug resistance phenomena.
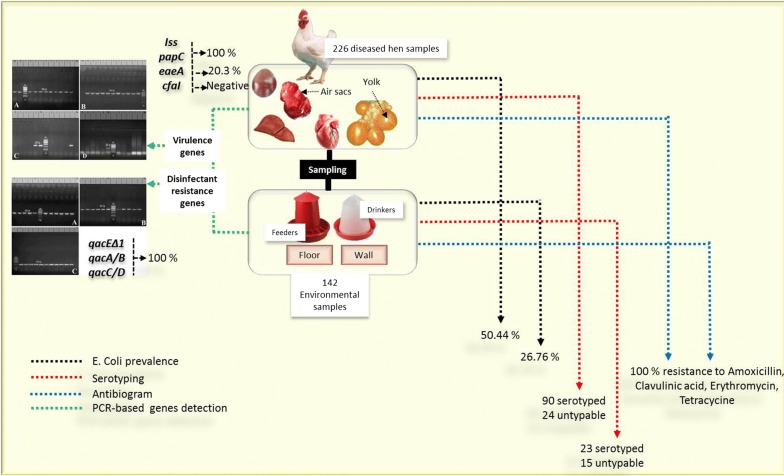

## Introduction

The rise of poultry production and industrial breeds of chicken, such as meat broilers, have been instituted as a method to promote gender equity, economic stability, and food security within many low and middle-income countries (LMICs). Despite the benefits, large-scale poultry production facilities within LMICs are often confronted with a tradeoff between animal welfare and addressing a growing economic demand, leading to high amounts of sub-therapeutic antibiotics for growth promotion and prophylaxis as well as disinfectant agents (Joint 2008; Udomsantisuk et al. 2011). High use of antibiotics and disinfectants could promote further antibiotic resistance (ARB) and disinfectant resistance (DR) (Eid et al. 2016).

One primary target for antibiotic and disinfect use is colibacillosis, which remains one of the major drivers of poultry morbidity and mortality, leading to severe losses (Barnes et al. 2008). These biosecurity risks can be further intensified as some *E. coli* avian diseases can be zoonotically transmitted via a trophic transmission (González-Zorn et al. 2005) or occupational exposure (Bisi-Johnson et al. 2011). Knowledge of *E. coli* serology can inform how to best treat diseases based upon typing categorizations. For instance, *E. coli* serotypes can cause intestinal illness with digestive signs, while other serotypes referred to as Avian Pathogenic *E. coli* belong to ExPEC that cause various symptoms in chicken either systemic or localized including; omphalitis, respiratory colibacillosis and colisepticemia (Rodriguez-Siek et al. 2005; Mellata 2013). The plasticity of *E. coli* pathogenicity is a result of an extensive range of virulence factors that are regulated and encoded by virulence determinant genes such as *(iss*, *papC*, *eaeA*, and *CFAI*) (De Carli et al. 2015; Eid et al. 2019). Studies have found links of co-selection for bacterial resistance from disinfectant and antibiotic use. Often, selection for resistance to antibiotics can inadvertently lead to drug resistance by movable genetic components (Noguchi et al. 2005; Chuanchuen et al. 2007). Additionally, these multimodal pathways for resistance can also promote increased pathogenicity in other species of bacteria through horizontal gene transfer. The quaternary ammonium compounds could be a major cause of the antibacterial cross-resistance development (Buffet-Bataillon et al. 2012). Various disinfectant resistant genes were recorded in multidrug-resistant pathogenic bacterial species (Zhang et al. 2015) including; *qacA/B, qacC/D, qacE,* and *qacG* genes (Correa et al. 2008). Often, in low-to- middle large-scale animal operations often apply high amounts of disinfectants and sub-therapeutic antibiotics to mitigate are chemical agents that used to kill microorganisms on inanimate instruments by various mechanisms as well as a wide spectrum of activity and potency (Fraise et al. 2013). Quaternary ammonium compounds are less toxic, non-irritating substances that are widely used to disinfect poultry farms environment (Bore et al. 2007). Many reports revealed a molecular relationship between *qac* genes and antibiotic resistance in certain pathogenic bacteria (Sidhu et al. 2001).

This study was aimed to investigate the prevalence of virulence genes *(iss, papC, eaeA*, and *CFAI*) and QACs resistance genes (*qacEΔ1, QacA/B*, and *QacC/D*) in *E. coli* strains originated from diseased broiler chicken and farm environment as well as monitoring of the antimicrobial susceptibility of the isolated strains.

## Materials and methods

### Sampling

A total of 368 specimens were collected aseptically from large scale farms [142 environmental samples: feeders (n = 32), drinkers (n = 32), walls (n = 36) and floors (n = 42) and 226 samples from diseased broiler chickens: heart (n = 70), liver (n = 82), lung (n = 12), yolk (n = 30), spleen (n = 16) and air sac (n = 16)] at Ismailia Governorate, Egypt. Average broiler chicken age was 7 weeks and the average weight was 1.8 kg. Handling of birds was performed according to the Animal Ethics Review Committee of Suez Canal University, Egypt. Samples were collected in the period from November 2016 until August 2017. The collected samples were prepared for bacteriological examination.

### Isolation and identification of *E. coli*

The collected specimens were inoculated in peptone water and then incubated at 37 °C for 24 h. A loopful from the incubated broth was streaked onto MacConkey′s agar and EMB plates and then incubated at 37 °C for 24 h. Suspected colonies were identified by microscopical examination, cultural characters as well as biochemical reactions as described by Quinn et al. (2011).

### Serotyping of *E. coli* strains

The isolates of *E. coli* were subjected to serotyping where somatic (O) antigen was investigated by slide agglutination test as described by Edwars and William (1972). Flagellar (H) antigen stereotyping was performed as described by Davies and Wray (1997).

### Antimicrobial susceptibility testing

*Escherichia coli* strains were tested against 12 antimicrobial agents (ampicillin, amoxicillin/clavulanic acid, erythromycin, gentamicin, neomycin, tetracycline, doxycycline, levofloxacin, norfloxacin, trimethoprim/sulphamethoxazole, sulphamethoxazole, and colistin sulphate) according to the methods described by NCCLS (2015) using disc diffusion technique. The susceptibility was determined according to the size of the inhibition zone. Multidrug resistance (MDR) was categorized for resistance to two or more unique antibiotic classes.

### PCR detection of virulence and disinfectant resistance genes of *E. coli*

*Escherichia coli* serotypes (n = 113; 23 environmental strains and 90 strains of avian origin) were tested for the detection of 4 virulence genes *(iss, papC, eaeA,* and *cfaI)* and 3 QACs resistance genes (*qacEΔ1, QacA/B*, and *QacC/D*) by using PCR. The DNA extraction was performed as described by the instructions of QIAamp DNA mini kit. The reaction volume includes (6 μl of the extracted DNA, 12.5 μl of Emerald Amp GT PCR master mix (2× premix) and 1 μl of each primer forward and reverse, PCR grade water 4.5 μl). Positive control strains were kindly given by Animal Health Research Institute, Dokki, Egypt. Primers used in PCR were illustrated in Table 1. PCR Protocol: initial denaturation at 94 °C for 5 min; denaturation at 94 °C for 30 s; annealing at 54 °C for 30 s for *iss* gene, at 51 °C for 30 s for *eaeA*, at 58 °C for 40 s for *papC*, at 50 °C for 40 s for *cfaI,* 58 °C for 40 s for qacE*Δ1* gene, at 53 °C for 40 s for *qacA/B* and at 53 °C for 30 s for *qacC/;* extension at 72 °C for 30 s in *iss*, *eaeA* and *qacC/D*, at 72 °C for 40 s in *papC* and *cfaI* qacE*Δ1* and *qacA/B*; cycles repeated for 35 times. Finally, the PCR products were separated by using electrophoresis and then photographed.

**Table 1:** Oligonucleotide primers sequences encoding for amplification of virulence genes and QACs resistance genes

Primer	Target gene	Primer sequence (5′–3′)	Product (bp)	References
*iss*-*1*	*Iss*	F-ATGTTATTTTCTGCCGCTCTG	266	Yaguchi et al. (2007)
*iss*-*2*		R-CTATTGTGAGCAATATACCC		
*eaeA*-1 (intimin)	*eaeA*	F-ATG CTT AGT GCT GGT TTA GG	248	Bisi-Johnson et al. (2011)
*eaeA*-*2*		R-GCC TTC ATC ATT TCG CTT TC		
*papC*-*1*	*papC*	F-TGATATCACGCAGTCAGTAGC	501	Jin et al. (2008)
*papC*-*2*		R-CCGGCCATATTCACATAA		
*CFAI*-*1*	*cfaI*	F-GCTCTGACCACAATGTTGA	364	Ghosal et al. (2007)
*CFAI*-*2*		R-TTACACCGGATGCAGAATA		
QacE*Δ1*-1	*qacEΔ1*	F-TAA CCCTACACAAATTGGGAGATAT	362	Chuanchuen et al. (2007)
QacE*Δ1*-2		R-GCC TCC GCA GCG ACT TCC ACG		
QacA/B-1	qacA/B	F-GCAGAAAGTGCAGAGTTCG	361	Noguchi et al. (2005)
QacA/B-2		R-CCAGTCCAATCATGCCTG		
QacC/D-1	*qacC/D*	F-GCCATAAGTACTGAAGTTATTGGA	195	
QacC/D-2		R-GACTACGGTTGTTAAGACTAAACCT		

### Statistical analyses

The data frequencies were analyzed by the nonparametric test (Chi square) with the aid of SAS (2004) software to test the null hypothesis of different treatment groups. The level of significance was P < 0.05.

## Results

### Prevalence of *E. coli* in diseased broiler chickens and farm environment

The bacteriological examination of 142 environmental samples revealed 38 *E. coli* strains (26.76%) including; feeders, drinkers, walls and floors samples with percentages of 37.5%, 31.25%, 11.11%, 28.57%, respectively. While 226 organ samples, revealed 114 *E. coli* strains with a prevalence of (50.44%). *E. coli* was isolated from internal organs (heart, liver, lung, yolk, spleen, air sac) with percentages of 42.86%, 60.98%, 33.33%, 20%, 50%, 100%, respectively. The total *E. coli* prevalence was (41.30%) as illustrated in Table 2.

**Table 2:** Total prevalence of *E. coli* isolated from all examined samples (feeder, drinker, wall, floor and organs of diseased broiler chickens)

Sources	Type of samples	No. of examined samples	*E. coli*		*Chi* square *P* value
			No.	%	
Environmental samples	Feeder	32	12	37.5	0.0792 NS
	Drinker	32	10	31.25	
	Wall	36	4	11.11	
	Floor	42	12	28.57	
Total		142	38	26.76	
Organs of diseased broiler chickens (ExPEC)	Heart	70	30	42.86	< 0.0001*
	Liver	82	50	60.98	
	Lung	12	4	33.33	
	Yolk	30	6	20	
	Spleen	16	8	50	
	Air sac	16	16	100	
Total		226	114	50.44	< 0.0001*
Total of all		368	152	41.30	

%The percentage was calculated according to the no. of each type of samples

### Serotyping of the isolated *E. coli* strains

In this study, 38 *E. coli* strains originated from environmental samples were subjected to serological identification, 23 strains (60.5%) have belonged to the following 10 different serotypes: O78 (13.16%), O119:H4, O113:H4, O169, O91:H21, O142, O111:H2, O1:H7, O26:H11 and O128:H2 (5.26% for each), while 15 strains (39.5%) were untypable. In addition, 114 *E. coli* strains (originated from organs of diseased broilers) were subjected to serological identification, 90 strains (78.95%) have belonged to the following 12 different serotypes: O1:H7 (13.16%), O78 (13.16%), O126 (8.77%), O91:H21 (8.77%), O125:H21, O44:H18, O121:H7, O15:H2, O146:H21, O124, O20 and O128:H2 (4.39% for each), moreover 24 strains (21.05%) were untypable as described in Table 3.

**Table 3:** Serotyping of *E. coli* strains isolated from environmental and diseased broiler chickens samples

Serotypes	Environmental *E. coli* (n = 38)		Organs of diseased broiler chickens (ExPEC) (n = 114)	
	No.	%	No.	%
O119:H4	2	5.26	–	–
O113:H4	2	5.26	–	–
O78	5	13.16	15	13.16
O169	2	5.26	–	–
O91:H21	2	5.26	10	8.77
O142	2	5.26	–	–
O111:H2	2	5.26	–	–
O1:H7	2	5.26	15	13.16
O26:H11	2	5.26	–	–
O128:H2	2	5.26	5	4.39
O126	–	–	10	8.77
O125:H21	–	–	5	4.39
O44:H18	–	–	5	4.39
O121:H7	–	–	5	4.39
O15:H2	–	–	5	4.39
O146:H21	–	–	5	4.39
O124	–	–	5	4.39
O20	–	–	5	4.39
Total	23/38	60.50	90/114	78.95
Untyped	15/38	39.50	24/114	21.05

* *Chi* square (P < 0.0243)

### Antimicrobial susceptibility of *E. coli* strains

As described in Table 4, the antimicrobial susceptibility testing of the isolated strains proved that, the tested strains were highly resistant (100%) to, ampicillin, erythromycin and tetracycline, followed by amoxicillin-clavulanic acid, norfloxacin, and sulphamethoxazole (80.92% for each), trimethoprim/sulphamethoxazole (75%) and gentamycin (50%). While (100%) of the tested strains were sensitive to colistin sulphate, followed by neomycin (87.5%). Meanwhile, the tested strains were intermediate sensitive to doxycycline (75%) and levofloxacin (62.5%).

**Table 4:** Results of antimicrobial susceptibility testing of the isolated *E. coli* strains (n = 152)

Antimicrobial disc	No. of *E. coli* (n = 152)					
	Resistant		Intermediate		Sensitive	
	No	%	No	%	No	%
Ampicillin	152	100	0	0	0	0
Amoxicillin/clavulanic acid	123	80.92	29	19.08	0	0
Erythromycin	152	100	0	0	0	0
Gentamicin	76	50	0	0	76	50
Neomycin	0	0	19	12.5	133	87.5
Tetracycline	152	100	0	0	0	0
Doxycycline	38	25	114	75	0	0
Levofloxacin	48	31.58	95	62.5	9	5.92
Norfloxacin	123	80.92	19	12.5	10	6.58
Trimethoprim/sulphamethoxazole	114	75	0	0	38	25
Sulphamethoxazole	123	80.92	0	0	29	19.08
Colistin sulphate	0	0	0	0	152	100
*Chi* square *P* value	< 0.0001*		< 0.0001*		< 0.0001*	

% calculated according to No of tested *E. coli* strains

### PCR detection of virulence genes and QACs resistance genes

**Fig. 1:**
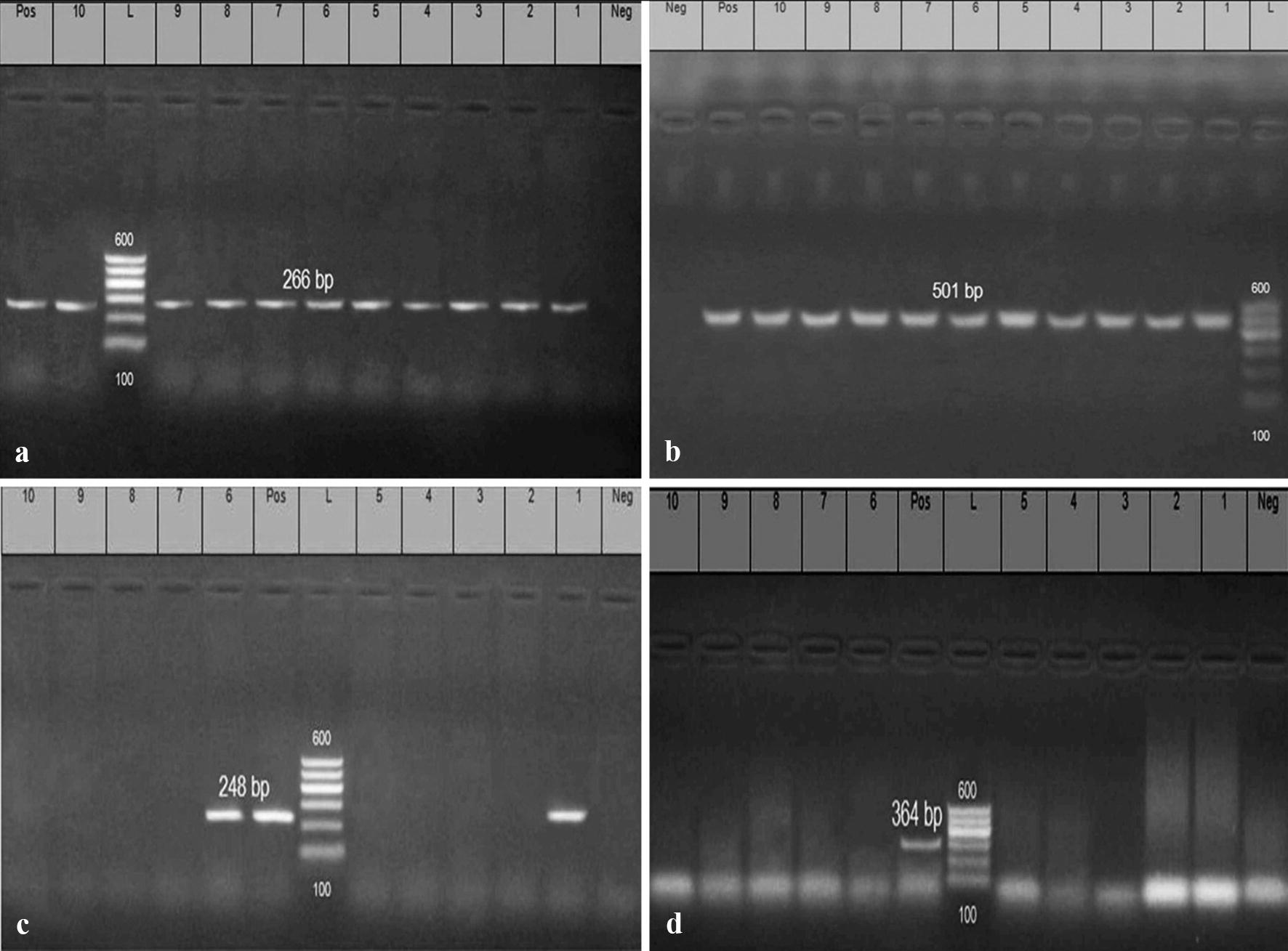
Electrophoretic pattern of PCR products of *E. coli iss, papC, eaeA,* and *cfaI* genes: **a** (L): the DNA molecular size ladder (Gelpilot 100 bp ladder); (Neg.): negative control; (Pos.): positive control; (lanes 1, 2, 3, 4, 5, 6, 7, 8, 9 and 10): positive amplification of 266 bp of *iss* gene of different *E. coli* strains. **b** (L): the DNA molecular size ladder (Gelpilot 100 bp ladder); (Neg.): negative control; (Pos.): Positive control; (lanes 1, 2, 3, 4, 5, 6, 7, 8, 9 and 10): positive amplification of 501 bp of *papC* gene of different *E. coli* strains. **c** (L): the DNA molecular size ladder (Gelpilot 100 bp ladder); (Neg.): negative control; (Pos.): Positive control; (lanes 1 and 6): positive amplification of 248 bp of *eaeA* gene; (lanes 3,4,5,7,8,9,10): Negative strains. **d** (L): the DNA molecular size ladder (Gelpilot 100 bp ladder); (Neg.): negative control; (Pos.): positive control; (lanes 1, 2, 3, 4, 5, 6, 7, 8, 9 and 10): negative amplification of 364 bp of *cfaI* gene of different *E. coli* strains

PCR was used for detection and amplification of 4 virulence genes (*iss, papC*, *eaeA* and *CFAI*) in the isolated strains as illustrated in Table 5, where (100%) of the tested strains were positive for *iss* gene at specific amplicon size 266 bp (Fig. 1a) and *papC* gene with specific amplicon size 501 bp (Fig. 1b). Only (20.3%) of the tested isolates were positive for *eaeA* gene with specified amplicon size 248 bp (Fig. 1c), the positive strains including; O1:H7 (n = 12), O78 (n = 5), O128:H2 (n = 2), O119:H4 (n = 2) and O113:H4 (n = 2). In addition, *cfaI* gene was absent in all examined strains as shown in (Fig. 1d).

**Table 5:** Prevalence of virulence genes and QACs resistance genes between the isolated *E. coli* serotypes

Sources	Type of sample	Serotypes	No of tested serotypes	*iss*	*papC*	*eaeA*	*cfaI*	*qacEΔ1*	*qacA/B*	*qacC/D*
Environmental *E. coli*	Feeder	O119:H4	2	2	2	2	0	2	2	2
		O113:H4	2	2	2	2	0	2	2	2
		O142	2	2	2	0	0	2	2	2
	Floor	O78	5	5	5	5	0	5	5	5
		O111:H2	2	2	2	0	0	2	2	2
		O1:H7	2	2	2	2	0	2	2	2
	Drinker	O26:H11	2	2	2	0	0	2	2	2
		O169	2	2	2	0	0	2	2	2
		O91:H21	2	2	2	0	0	2	2	2
	Wall	O128:H2	2	2	2	2	0	2	2	2
Organs of diseased broiler chickens	Heart	O15:H2	5	5	5	0	0	5	5	5
		O78	7	7	7	0	0	7	7	7
		O91:H21	5	5	5	0	0	5	5	5
		O124	5	5	5	0	0	5	5	5
		O146	5	5	5	0	0	5	5	5
	Liver	O126	10	10	10	0	0	10	10	10
		O44:H18	5	5	5	0	0	5	5	5
		O1:H7	15	15	15	10	0	15	15	15
		O125:H21	5	5	5	0	0	5	5	5
		O78	8	8	8	0	0	8	8	8
		O91:H21	5	5	5	0	0	5	5	5
	Spleen	O121:H7	5	5	5	0	0	5	5	5
	Air sac	O20	5	5	5	0	0	5	5	5
		O128:H2	5	5	5	0	0	5	5	5
Total			113	113	113	23	0	113	113	113

**Fig. 2:**
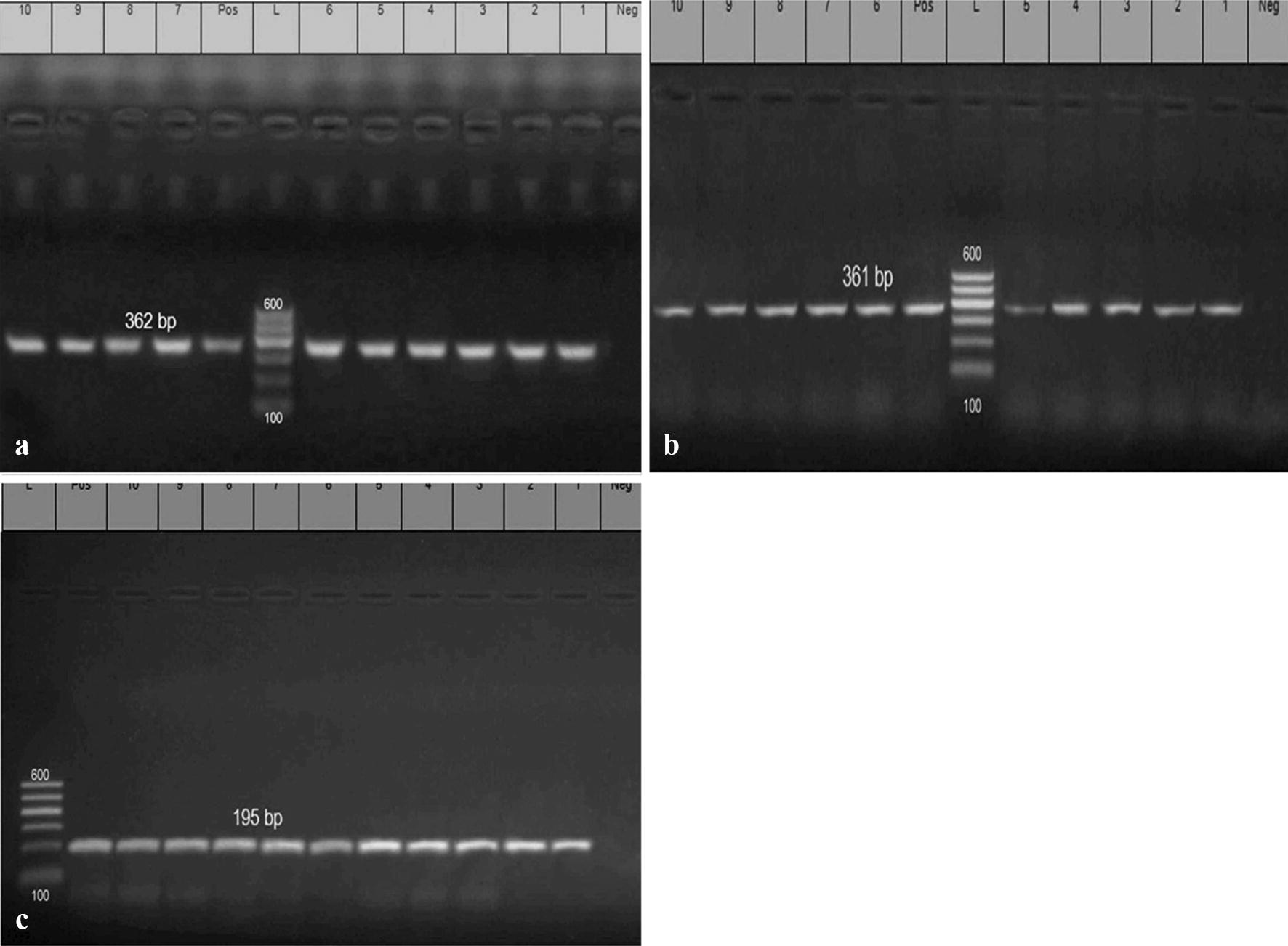
Electrophoretic pattern of PCR products of *E. coli qacEΔ1, QacA/B*, and *QacC/D* genes: **a** (L): the DNA molecular size ladder (Gelpilot 100 bp ladder); (Neg.): negative control; (Pos.): positive control; (lanes 1, 2, 3, 4, 5, 6, 7, 8, 9 and 10): positive amplification of 362 bp of *qacEΔ1* gene of different *E. coli* strains. **b** (L): the DNA molecular size ladder (Gelpilot 100 bp ladder); (Neg.): negative control; (Pos.): positive control; (lanes 1, 2, 3, 4, 5, 6, 7, 8, 9 and 10): positive amplification of 361 bp of *qacA/B* gene of different *E. coli* strains. **c** (L): the DNA molecular size ladder (Gelpilot 100 bp ladder); (Neg.): negative control; (Pos.): positive control; (lanes 1, 2, 3, 4, 5, 6, 7, 8, 9 and 10): positive amplification of 195 bp of *qacC/D* gene of different *E. coli* strains

Concerning the detection of the QACs resistance genes, PCR was used for the detection and amplification of (*qacAΔ1, qacA/B* and *qacC/D*) genes in the isolated strains, all the tested strains (100%) were positive for *qacAΔ1, qacA/B* and *qacC/D* genes with specific amplicon size 362 bp, 361 bp and 195 bp, respectively, as illustrated in Fig. 2a–c and Table 5.

## Discussion

Cross-resistance between antibiotics and QAC could occur by various mechanisms on the same resistance plasmid and or transposon (Hegstad et al. 2010). The presence of Quaternary Ammonium Compounds determinants on various mobile constituents helps in the transport of resistance to another microorganism (Gillings et al. 2009). The massive and improper application of antibiotics for long-term in poultry farms resulted in multidrug resistance in different bacterial pathogens (Singer and Hofacre 2006). In the present work, the bacteriological examination showed that the prevalence of *E. coli* in environmental specimens was (26.76%), while was (50.44%) in diseased broiler chickens. The total prevalence of *E. coli* was (41.30%) as illustrated in Table 2. The prevalence of *E. coli* from different environmental sources is not significantly differed (P > 0.05). Meanwhile, higher significant differences were recorded (P < 0.0001) in the prevalence of *E. coli* from organs of diseased broiler chickens compared to those of the environmental sources. The highest prevalence was recorded in air sac (100%) followed by liver (60.98%), while the lowest prevalence was recorded in the yolk (20%). Higher prevalence of (84%) was obtained by Oboegbulem et al. (2009) who isolated this organism from commercial and backyard poultry farms and chicken markets. Multiple predisposing conditions could rise the susceptibility of poultry to colibacillosis, including; respiratory viruses, overcrowding, bad handling of birds and bad sanitation (Eid et al. 2016).

Regarding the serotyping of the isolated *E. coli* strains, 38 *E. coli* strains isolated from environmental samples were subjected to the serological identification, 23 strains (60.5%) have belonged to 10 serogroups and the most predominant serogroup was O78 (13.16%), while 15 *E. coli* strains (39.5%) were untypable. In addition, 114 *E. coli* strains originated from organs of diseased broilers were subjected to serological identification, 90 strains (78.95%) have belonged to 12 different serogroups and the most predominant serogroups were O1:H7 (13.16%), O78 (13.16%), O126 (8.77%), O91:H21 (8.77%), while 24 strains (21.05%) were untypable, as described in Table 3. There is a significant difference in the prevalence of the isolated serotypes (P < 0.0243). These results disagree with those obtained by Yousef et al. (2015) who recorded that the most prevalent serotypes originated from different sources of poultry broiler farms were untypable *E. coli* serovars; followed by O26; then O2, O124, O125, and O114. Chart et al. (2000) proved that the Avian Pathogenic *E. coli* are mainly included specific serotypes, especially serotypes O78, O2 and O1, followed by O55, and O15. The emergence of certain serotype and its responsibility for disease occurrence is mainly depending upon the health condition of chicken, the environmental conditions, handling and management procedures (Srinivasan et al. 2013).

In the present study, the isolated *E. coli* strains were tested against 12 antimicrobial agents, The resistance and sensitivity of the isolated strains to different antimicrobial discs were differed significantly (P < 0.0001) as described in Table 4. The tested strains showed multiple drug resistance and were highly resistant (100%) to ampicillin, erythromycin and tetracycline, followed by amoxicillin/clavulanic acid, norfloxacin, and sulphamethoxazole (80.92%), trimethoprim/sulphamethoxazole (75%) and gentamycin (50%). While (100%) of the tested strains were sensitive to colistin sulphate, followed by neomycin (87.5%). These results are nearly agreed with those obtained by Hashem et al. (2012) and Ola (2017).

Production of β -lactamase enzyme that breaks down the beta-lactam ring of penicillin is the major mechanism of antibiotic resistance in *E. coli*. Gene encoding β -lactamase enzyme could be carried on plasmid or on bacterial chromosome (Udomsantisuk et al. 2011), while aminoglycosides resistance is mainly taking place in pathogenic *E. coli* due to aminoglycoside modifying enzyme (Galimand et al. 2003) which is encoded on R-plasmids (González-Zorn et al. 2005). Sulfonamides, penicillins and tetracyclines, and are the most popular and the oldest antimicrobial agents that used heavy against bacterial infection so that a high level of drug resistance has emerged with the time (Joint 2008).

Regarding the genetic detection of virulence genes, in the present study PCR was used for detection and amplification of (*iss, papC*, *eaeA*, and *cfaI*) genes, as illustrated in Table 5 (100%) of the tested strains were positive for *iss* gene at specific amplicon size 266 bp (Fig. 1a) and *papC* gene with specific amplicon size 501 bp (Fig. 1b). Only (20.3%) of the tested strains were positive for *eaeA* gene with specified amplicon size 248 bp (Fig. 1c), furthermore, all the examined strains were negative for *cfaI* gene as illustrated in (Fig. 1d). These results are agreed with those described by Dissanayake et al. (2014) who reported that 85.4% of Avian Pathogenic *E. coli* which originated from diseased birds suffering from colibacillosis in the USA were positive for *iss* gene, also this result is supported by Ewers et al. (2004) who mentioned that *iss* gene was detected in 82.7% of APEC strains originated from birds suffering from coli septicemia in Germany. In contrary to the results of *papC* gene in this study Rodriguez-Siek et al. (2005) stated that the *papC* gene is commonly associated APEC with a percentage (44.1%).

Moreover, attaching and effacing is a term that used to clarify the lesion caused by *E. coli* in the host intestine, where (attaching) refers to the intimate adhesion to the cell membrane of intestinal cells, while (effacing) refers to the destruction of intestinal microvilli (Stordeur et al. 2000). In this study, the prevalence of *eaeA* gene was (20.3%), these results are agreed with Ola (2017) who reported that the incidence rate of *eaeA* gene in the tested *E. coli* strains was 15.79%. Also, these results nearly agreed with those obtained by (DebRoy and Maddox 2001). In contrary to these findings, Ramadan et al. (2016) stated that all the tested *E. coli* strains derived from chicken viscera were carried the *eae* gene (100%).

Concerning the detection of the disinfectant resistance genes, PCR was used for detection and amplification of QACs resistance (*qacAΔ1, qacA/B, qacC/D*) genes in the isolated *E. coli* strains, as illustrated in Table 5 all the tested *E. coli* strains (100%) were positive for *qacAΔ1, qacA/B* and *qacC/D* genes with specific amplicon size 362 bp, 361 bp and 195 bp, respectively, as illustrated in (Fig. 2a–c). These results are supported by Amira (2016) who found that the distribution of *qacEΔ1* was (93.1%). The massive use of QACs in the farm environment could result in acquired QACs resistance in *E. coli* strains (Sidhu et al. 2002). Many QACs resistant genes are commonly associated with multidrug-resistant pathotypes especially *qacC/D*, *qacA/B*, and *qacE* (Zhang et al. 2015). The *qacEΔ1*gene is common in enteric bacterial pathogens possessing Sulphonamide resistant determinants. Seventy percent of *qacEΔ1*- +ve strains exhibit cross-resistance to Sulphamethoxazole, 60% of *qacEΔ1*- +ve strains exhibits cross-resistance to Sulfamethoxazole-trimethoprim. Also, 40% of *qacEΔ1*- +ve strains were highly resistant to Gentamicin (Kücken et al. 2000).

In conclusion, *E. coli* continues to be one of the most important pathogens in poultry and poultry farm environment, the most predominant *E. coli* serotypes affecting broiler chickens are O78, O1:H7, O91:H21 and O126. The QACs resistance genes are frequently distributed with the multidrug-resistance pathotypes which may be transmitted to humans by the consumption of chickens or any byproduct containing such strains. The high proportion of virulence genes (*iss* and *papC*) and the multidrug-resistance phenomena is prevalent in Avian Pathogenic *E. coli* and environmental strains. There is a directly proportional relationship between the presence of multidrug-resistance, disinfectant resistant genes, virulence genes and the severity of lesions associated with *E. coli* infection and complications.

## Data Availability

Not applicable.
